# Spatial ecology of a range‐expanding bumble bee pollinator

**DOI:** 10.1002/ece3.4722

**Published:** 2019-01-08

**Authors:** Liam P. Crowther, David J. Wright, David S. Richardson, Claire Carvell, Andrew F. G. Bourke

**Affiliations:** ^1^ School of Environmental Sciences University of East Anglia, Norwich Research Park Norwich UK; ^2^ School of Biological Sciences University of East Anglia, Norwich Research Park Norwich UK; ^3^ NERC Centre for Ecology & Hydrology Crowmarsh Gifford Wallingford UK

**Keywords:** colonization, foraging distance, genetic structure, invasive species, microsatellite, population biology

## Abstract

Molecular methods have greatly increased our understanding of the previously cryptic spatial ecology of bumble bees (*Bombus* spp.), with knowledge of the spatial ecology of these bees being central to conserving their essential pollination services. *Bombus hypnorum, *the Tree Bumble Bee, is unusual in that it has recently rapidly expanded its range, having colonized much of the UK mainland since 2001. However, the spatial ecology of *B. hypnorum* has not previously been investigated. To address this issue, and to investigate whether specific features of the spatial ecology of *B. hypnorum* are associated with its rapid range expansion, we used 14 microsatellite markers to estimate worker foraging distance, nest density, between‐year lineage survival rate and isolation by distance in a representative UK *B. hypnorum* population. After assigning workers to colonies based on full or half sibship, we estimated the mean colony‐specific worker foraging distance as 103.6 m, considerably less than values reported from most other bumble bee populations. Estimated nest density was notably high (2.56 and 0.72 colonies ha^−1^ in 2014 and 2015, respectively), estimated between‐year lineage survival rate was 0.07, and there was no evidence of fine‐scale isolation by distance. In addition, genotyping stored sperm dissected from sampled queens confirmed polyandry in this population (mean minimum mating frequency of 1.7 males per queen). Overall, our findings establish critical spatial ecological parameters and the mating system of this unusual bumble bee population and suggest that short worker foraging distances and high nest densities are associated with its rapid range expansion.

## 1 INTRODUCTION

Bumble bees (*Bombus* spp.) are key pollinators of many wild plants (Ollerton, Winfree, & Tarrant, 2011) and economically important crops (Garratt et al., 2014). Along with those of other insects, their pollination services support food security (Gill et al., 2016; Klein et al., 2007) and account for around 10% of global agricultural production (Gallai, Salles, Settele, & Vaissière, 2009). Across both Europe and North America, many bumble bee species are declining, with potential reasons ranging from habitat loss and fragmentation to impacts of pesticides and pathogens (Cameron et al., 2011; Potts et al., 2010; Williams & Osborne, 2009). However, some bumble bee species are expanding their ranges due either to deliberate introduction (Schmid‐Hempel, Schmid‐Hempel, Brunner, Seeman, & Allen, 2007; Schmid‐Hempel et al., 2014) or to natural colonization of areas outside their native ranges (Owen et al., 2012). Understanding the elements of bumble bee spatial ecology, including foraging ecology, that underpin these patterns of both decline and range expansion is critical to developing effective management and conservation strategies for long‐term population persistence in this threatened group.

A notable example of a range‐expanding bumble bee population is the UK population of the Tree Bumble Bee, *Bombus hypnorum*. It was first recorded in southern England in 2001 (Goulson & Williams, 2001). While it could have colonized naturally or arrived by accidental human‐mediated transportation (Williams, Lobo, & Meseguer, 2018), *B. hypnorum *has rapidly expanded its range within the UK (by 900 km since 2001) and now occurs throughout England and Wales and in much of Scotland (Bates et al., 2011; Crowther, Hein, & Bourke, 2014; Jones & Brown, 2014). Hence, unlike populations of other widespread species of bumble bee in the United Kingdom, whose ranges have remained stable on a UK‐wide scale (Macdonald, 2001), the UK *B. hypnorum* population has greatly increased. However, the spatial ecology of *B. hypnorum* has not been previously investigated, either in the United Kingdom or in its original range in continental Europe and Asia.

In recent years, molecular methods have provided valuable tools with which to estimate parameters of the spatial ecology of bumble bees in wild populations (reviewed by Woodard et al., 2015). An important finding is that worker foraging distance (the distance that workers fly from their nest to forage at plants for nectar and pollen) is plastic with respect to resource availability. Specifically, colony‐specific worker foraging distance falls as the proportion of high‐quality foraging habitat in the neighborhood increases (Carvell et al., 2012; Jha & Kremen, 2013; Pope & Jha, 2018; Redhead et al., 2016), presumably because in resource‐rich conditions workers save energetic costs by foraging over shorter distances (Williams, 1989). This finding suggests that worker foraging distance and other aspects of spatial ecology are not simply autecological traits (i.e., species‐level traits) but are instead best understood as functions of the density and spatial arrangement of resources in the landscape. Molecular methods have also shown that, in wild bumble bee populations, a higher density of floral resources around individual nests is linked to daughter queens surviving to the spring emergence stage in the following year at higher frequency, that is, greater lineage survival (Carvell et al., 2017). Therefore, an emerging synthesis suggests that high‐quality resources at sufficient densities can lead to reduced worker foraging distances and enhanced rates of queen survivorship and hence, by inference, enhanced rates of population increase. If correct, this synthesis predicts that in rapidly expanding populations, which are inferred to be characterized by colonies exhibiting a high productivity of new queens, bumble bee colonies should encounter resources at densities that facilitate short worker foraging distances.

In this study, we therefore used molecular methods, based on the reconstruction of foraging workers' colony membership using neutral genetic markers (microsatellites), to investigate the spatial ecology of the UK *B. hypnorum* population. Our overall aim was to estimate key spatial ecological parameters in this population, including worker foraging distance, nesting density, between‐year lineage survival rate and fine‐scale isolation by distance, and investigate whether specific features of the spatial ecology of *B. hypnorum* are associated with its rapid range expansion in the United Kingdom. In particular, we sought to test whether *B. hypnorum* exhibits the short worker foraging distances expected in a rapidly expanding population. Our study landscape was a typical suburban landscape in southern England (in Norwich, UK). This was selected because, across urban‐rural gradients in southern England, *B. hypnorum *workers occur much more frequently in suburban landscapes (Bates et al., 2011; Crowther et al., 2014), so suggesting that suburban and similar habitats are representative of those facilitating the population increase that underlies *B. hypnorum*'s UK range expansion.

Unlike the queens of most species of bumble bee, which mate singly, *B. hypnorum *queens are facultatively polyandrous, with studies in continental Europe estimating a mating frequency of 1–6 males per queen (Brown, Schmid‐Hempel, & Schmid‐Hempel, 2003; Estoup, Scholl, Pouvreau, & Solignac, 1995; Paxton et al., 2001; Schmid‐Hempel & Schmid‐Hempel, 2000). The degree of polyandry (percentage of polyandrous queens) varies geographically across populations from 0% to 67% (Estoup et al., 1995; Schmid‐Hempel & Schmid‐Hempel, 2000; Paxton et al., 2001; Brown, Schmid‐Hempel & Schmid‐Hempel 2003). Hence, we also sought to characterize the mating system of the UK *B. hypnorum* population. This was both because the mating system is a fundamental aspect of the social and reproductive biology of eusocial insects and because the level of polyandry affects the assignment of workers to colonies based on genetic markers. In particular, under facultative polyandry, nest‐mate workers may include both full sisters and half‐sisters (i.e., maternal but not paternal sisters), and a priori information on the level of polyandry is needed to validate and inform the assignment of half‐sisters (Wang, 2004; Wang & Santure, 2009).

Overall, therefore, we addressed the following four research questions: (a) What is the mating system of the UK *B. hypnorum* population and, specifically, what is the frequency distribution of male mate number and the mean mating frequency per queen? (b) What is the worker foraging distance and is it short as predicted? (c) What are the nesting density and between‐year lineage survival rate of *B. hypnorum *in the study landscape? (d) Does *B. hypnorum *exhibit fine‐scale spatial genetic structuring (isolation by distance)?

## 2 METHODS

### 2.1 Worker and queen sample collection

*Bombus hypnorum *workers were collected from a 2 × 2 km (400 ha) sampling area on the western edge of Norwich, Norfolk, UK (Supporting information Figure S1). *B. hypnorum *was first recorded in the 10 × 10 km grid square enclosing the sampling area in 2008 (Bees Wasps & Ants Recording Society, 2016). The sampling area comprised a mix of suburban residential areas, parks, woodland, semi‐natural areas and university campus that are typical of lowland, non‐agricultural habitat within the United Kingdom. Workers were sampled in two successive summers in the periods 15 May 2014 – 16 June 2014 and 28 May 2015 – 1 July 2015. Within a given year, these dates straddle the peak of observed worker abundance for *B. hypnorum* for the locality (Crowther et al., 2014). To distribute sampling evenly, the area was split into 16 equal 500 × 500 m (25 ha) divisions, hereafter “sampling squares” (Supporting information Figure S1). In each year, *B. hypnorum *workers were sampled by free‐searching all the publicly accessible suitable habitat within every sampling square. A net was used to capture all encountered workers (while foraging at flowers or free‐flying) until either 40 workers had been caught from a given sampling square or three 2‐hr searches on separate days had been completed. Sampling took place during dry weather when air temperature was 15°C or higher, during the hours 1000–1700.

Tissue for DNA extraction was non‐lethally sampled by temporarily restraining the worker and clipping the tarsal tip of a mid‐leg (Holehouse, Hammond, & Bourke, 2003). Each tarsal tip was then stored individually in 100% ethanol in a 1.5‐ml tube at ambient temperature until extraction. The exact sampling location of each specimen was recorded using a Garmin eTrex handheld GPS receiver, with an accuracy of approximately 4 m.

To characterize their mating system, whole *B. hypnorum *queens were collected from five sites. All sites were public parks selected for their high density of early‐season flowering plants and lay within 10 km of the worker sampling area (Supporting information Table S1). All queen sampling took place during the periods 1 March 2014 – 20 April 2014 and 3 March 2015 – 21 April 2015. Each site was searched freely for 2–4 hr, and all encountered queens were captured and killed by freezing at −20°C, after which they were kept frozen until dissection.

### 2.2 Sperm sample collection by spermathecal dissection

Queens were dissected under a stereomicroscope at 40× magnification to isolate the spermatheca (Supporting information Text S1; Figure S2). The spermatheca was suspended in a small drop of distilled water on a slide and manipulated with fine needles to separate the mass of stored sperm from any spermathecal (queen) tissue as completely as possible (Supporting information Figures S2c,d). To minimize contamination, tools and slides were cleaned with bleach between dissections. Dissections were carried out in batches of five, with a negative control in each batch prepared using the same tools, slides and water source to isolate a droplet of the distilled water. DNA extraction from the isolated sperm and from the negative control samples was performed immediately after dissection. Wing muscle was also dissected from each queen, to provide tissue to determine the queen's own genotype.

### 2.3 DNA extraction and genotyping

DNA was extracted using a modified ammonium‐acetate ethanol precipitation (Richardson, Jury, Blaakmeer, Komdeur, & Burke, 2001) from worker (tarsal tip), queen (wing muscle) and sperm (isolated from spermathecae) samples. Tarsal tips were first frozen using liquid nitrogen for 2 min and crushed to a powder before digestion. To maximize DNA yield, the ethanol precipitation step included incubation at −20°C for 3 hr. Extracted DNA was suspended in low‐T.E. buffer (10 mM Tris.HCL, 0.1 mM EDTA) and stored at −20°C.

Twenty‐four microsatellite primer pairs, previously characterized from other *Bombus *species (Estoup et al., 1995; Reber Funk, Schmid‐Hempel, & Schmid‐Hempel, 2006; Stolle et al., 2009), were tested on DNA from 10 to 20 worker tarsal tips to ascertain whether they amplified polymorphic loci in *B. hypnorum*. This test yielded 20 polymorphic microsatellite markers. The four remaining loci were monomorphic or failed to amplify and were excluded (Supporting information Table S2). For PCR, the 20 selected polymorphic loci were divided into three multiplexes (Supporting information Table S3), designed using Multiplex Manager v1.2 (Hollely & Geerts, 2008). The minimum distance between same‐dye markers was 14 base pairs and the complementarity threshold was set to 7 base pairs (Supporting information Table S3). PCR was carried out in 2 μl reaction volumes. Up to 15 ng of sample DNA was added to each reaction well, where extraction yields permitted. As expected, the DNA yields of extractions from sperm were considerably lower than those from worker and queen tissue. For sperm samples, one‐eighth of the total extraction yield was used as a template for each of the three multiplex PCRs. The DNA was dried at 50°C prior to the addition of aqueous reagents. Each reaction contained 1 μl of Qiagen Multiplex Master Mix (Qiagen, Manchester, UK) and 1 μl of primer mix with primer pairs at 0.08–0.50 M concentrations for queen and worker samples (Supporting information Table S3). All reagent volumes were doubled for PCRs using template DNA from sperm samples, as sample DNA concentrations were lower. Each reaction volume was covered with a droplet (ca. 10 μl) of mineral oil to prevent evaporation. In addition to the dissection controls described above, each plate included (a) a negative control for the reaction, consisting of all the reagents and primers but no template DNA, and (b) two positive controls using DNA from *B. hypnorum* queens whose multi‐locus genotype had been ascertained using multiple single‐locus PCRs.

For queen and worker samples, amplification conditions comprised the following: an activation step for 15 min at 95°C; 30 cycles of 30 s at 94°C, annealing for 90 s at 50°C and 1 min at 72°C; with a final extension of 5 min at 72°C. In order to increase peak height, the number of cycles was increased to 45 for sperm samples, but PCR conditions were otherwise identical.

PCR products were visualized using a 48‐well capillary ABI 3730 DNA analyser and a ROX‐500 internal size standard (Applied Biosystems). Fragments were sized using GeneMapper 4.0 software (Applied Biosystems, Paisley, UK). Alleles were only accepted when confirmed in two or more individuals. Extracted DNA from 80–120 workers (i.e., 12%–19% of samples) were re‐genotyped for each of the three multiplexes, repeating the PCR and analysis steps for 1,880 locus‐level genotypes, covering all loci. These data were used to calculate locus‐specific allelic mistyping rates. The per‐locus mean (range) allelic mistyping rate was found to be 2.26% (0.91%–3.17%). No negative controls contained peaks that corresponded to any amplified alleles. Four workers that had peaks corresponding to three alleles at one or more loci were excluded from further analyses, as it was not possible to determine whether they were triploid or whether original samples were contaminated. In total, 44 queens and their corresponding sperm samples and 645 workers (375 from 2014 and 270 from 2015) were sampled and genotyped.

### 2.4 Analysis of Hardy–Weinberg equilibrium, null allele frequencies and linkage disequilibrium

The genotypes of all worker samples were tested for deviations from Hardy–Weinberg equilibrium (HWE) within both years, corrected for multiple comparisons, using the R package “adegenet” (Jombart, 2008). Years were treated as separate subpopulations because, due to the recent colonization of the sampling area and surrounding area, it is possible that the local population was structured temporally. These analyses used all workers, some of which would have been full or half‐sisters. This should not have introduced bias (as offspring genotypes represent a random sample of parental genotypes) but would instead have made the test more conservative by inflating degrees of freedom. The frequencies of null alleles for all loci were estimated using the program Cervus 3.0 (Kalinowski, Taper, & Marshall, 2007). Pairwise tests for linkage across all combinations of the twenty loci were implemented using functions from the R package “pegas” (Paradis, 2008). To meet the assumptions of the analysis, loci were excluded from the colony assignment (below) if they exhibited one or more of: (a) significant deviation from HWE in both years after correction for multiple comparisons; (b) null allele frequencies in excess of 0.1; or (c) significant linkage with another, more informative locus, after correction for multiple comparisons. All data handling and statistical analysis were executed in R version 3.1.2 unless otherwise stated (R Development Core Team 2011).

### 2.5 Mating frequency of queens

We estimated the number of males that contributed to each sperm sample within each mated queen by comparing the genotypes in the sperm sample to the previously identified genotype of the queen from which the spermatheca was dissected. Males were assumed haploid because, although diploid males may occur in UK *B. hypnorum* populations (Jones & Brown, 2014), male diploidy in *Bombus* appears associated with sterility (Duchateau & Mariën, 1995). Although care was taken during the spermathecal dissections to separate the sperm from queen tissue, contamination of the sperm samples with queen tissue cannot be excluded. Therefore, if the sperm sample genotype contained any allele shared with the corresponding queen (hereafter, a “shared allele”), we assessed whether the allele was more likely to have originated from the sperm or the queen. For this purpose, we made two assumptions. First, if a shared allele arose from queen contamination, then both the queen's alleles for that locus (assuming the queen was a heterozygote) should have amplified and been present in the sperm sample genotype. Therefore, a shared allele that was not accompanied in the sperm genotype by an allele identical to a heterozygote queen's other allele at that locus was deemed to be a true male allele. Second, we assumed that queen contamination, if present, would result in a higher frequency of shared alleles than would be expected by chance, given random mating. This assumption was applied using our independent data regarding the queen genotypes (from genotyping the queen wing muscle samples) and the population allele frequencies (from genotyping the worker tarsal tip samples). We used these data in a simulation to identify which sperm samples were likely to be have been contaminated. These were considered to be samples in which the alleles of the corresponding queen appeared at a rate across loci higher than would be expected by chance, assuming that her mate(s) shared the same allele(s).

To perform the simulation (Simulation 1), we calculated, for every locus of every queen, assuming double mating (the commonest mating frequency of polyandrous *B. hypnorum* queens [Estoup et al., 1995; Schmid‐Hempel & Schmid‐Hempel, 2000; Paxton et al., 2001]), the probability that her alleles at that locus would match (i.e.. be the same as) the combined alleles of her two mates at that locus. We ran 10,000 Bernoulli trials of each of these probabilities and, within queens across loci, counted the number of matches. For each queen, the mean number of matching loci across the 10,000 replicates is hereafter referred to as the “expected number of matches” and, when divided by the number of loci for that queen, gives the “expected rate of matching.” The mean and variance across queens of the expected rate of matching were then used to calculate a critical value equal to the mean plus two standard deviations. Any sperm sample that matched its corresponding queen sample's genotype at a proportion of loci larger than the critical value was assumed to be contaminated, because matching the queen's genotype at such a high rate would be unlikely due to chance (assuming the normal distribution, *p* = 0.0228 when z = 2).

For each queen, we then estimated the minimum number of males with which she had mated as the greatest number of alleles per locus from her corresponding sperm sample that (a) were not attributable to the queen (i.e., that had not been identified as representing contamination as described above) and (b) were supported across two or more loci. Confirmation at two or more loci was required to limit the potential effect of any genotyping errors in the sperm samples as it was not possible to estimate error rates with these samples due to the limited DNA yields. These estimates, averaged across all 44 queens, provided a conservative estimate of the mean (per queen) mating frequency.

In order to estimate the uncertainty around this mean, a further simulation was then constructed (Simulation 2). For this, sampled queen genotypes were combined with simulated male genotypes, randomly generated using the population allele frequencies of the workers. The simulated “true” number of matings was allowed to vary from 1 to 9. Each queen genotype was then paired with 10,000 replicates of simulated sperm genotypes based on each “true” number of males. The number of male mates of each queen was then counted using a procedure identical to the one described earlier for actual sperm samples. This allowed us to estimate the probability of counting an observed number of males in the sperm samples given the simulated “true” number. These probabilities allowed us to infer the range of actual mating frequencies that could have led to the observed pattern of mating frequencies.

### 2.6 Colony assignment of workers and queens

The program COLONY v2 (Jones & Wang, 2010) was used to assign sampled workers to colonies on the basis of being full or half‐sisters (i.e., maternal but not paternal sisters). Following Dreier et al. (2014), the inclusion probability was set at ≥0.8. *Bombus *species exhibit an annual colony cycle, so workers sampled in one year cannot be full or half‐sisters of workers sampled the following year (even if there is bivoltinism, i.e., two colony cycles per year). Hence, workers were only assigned with workers sampled in the same year. The male mating system was specified as monogamous, again following Dreier et al. (2014), and the female mating system was specified as polygamous. As COLONY v2 does not allow female mating frequency to be specified directly, it was specified indirectly by setting prior values on the relative sizes of maternal and paternal sisterhoods. For a population with female mating frequency *m*, for every *n* offspring who share the same father, on average *mn* offspring will share the same mother. Under the assumption that matrilines and patrilines are sampled independently at rates based on their frequency in the population, our sample should therefore contain *mn* maternal sisters for every *n* paternal sisters. To estimate *n*, workers were initially assigned to colonies without using a priori information on the queen mating frequency, and, based on these assignments, the average size of a full sisterhood was then calculated. The size of full sisterhoods was taken to be reliably estimated by this procedure, as, under haplodiploidy, full sisters will always share a single paternal allele and have one of only two maternal alleles. Consequently, full sisters should be assigned with high accuracy compared to half‐sisters. This estimate of *n*, along with the value of *m* estimated above, were used within COLONY v2 to set priors of weight 0.25 on the expected size of sampled maternal (*mn*) and paternal (*n*) sisterhoods. This procedure followed recommendations within COLONY v2 for when the level of confidence in a priori knowledge of the mating frequency is relatively low (Jones & Wang, 2010). With respect to workers that were assigned to families with multiple patrilines, a maximum number of patrilines per colony was set using the range of individual mating frequencies returned by Simulation 2 (above). Reconstructed colonies were only accepted if they contained fewer than the maximum number of patrilines. As an additional test of the robustness of the colony reconstructions, we tested whether, across the worker sample as a whole, the pairwise distance between the sampling locations of reconstructed full sisters was significantly different from that of reconstructed half‐sisters. If half‐sisters were reconstructed with appreciably greater error, then this distance should have been greater for half‐sisters than for full sisters, because reconstructed half‐sisters would then have included more workers that were not in fact from the same colony.

Lastly, to determine whether any of the 44 collected queens were full or half‐sisters, a COLONY v2 analysis identical to the one used to assign workers to colonies was performed on the queens' genotypes at the same loci as those used in the worker analysis.

### 2.7 Colony‐specific worker foraging distance

To estimate colony‐specific worker foraging distance, we first estimated the most likely nest location of each reconstructed colony. This was done by calculating the mean centre of the GPS‐determined locations at which all workers assigned to a given colony were sampled. All colonies represented by two or more workers with sampling locations separated by more than 4 m (i.e., the precision of the GPS receiver) were used in this analysis, although this resulted in no further exclusions of accepted colonies. The mean‐centre approach followed that of Redhead et al. (2016) except that predicted colony locations were not “snapped” to nearby land cover types suitable for nesting. Since *B. hypnorum *frequently nests in above‐ground cavities, including in buildings (Benton, 2006), all of the study area was considered suitable *B. hypnorum* nesting habitat. The Euclidean distance between the location of each sampled worker and its estimated nest location was then calculated. The colony‐specific worker foraging distance was estimated as the mean of these distances for all workers assigned to a given colony. To investigate whether the size of the sampling area may have biased estimates of worker foraging distance the distribution of pairwise distances between full and half‐sisters was compared to that of unrelated workers.

### 2.8 Nesting density and lineage survival

Estimating nest density from colony assignments based on genetic markers requires an estimate of the number of nests not represented in the sample (Chapman, Wang, & Bourke, 2003; Darvill, Knight, & Goulson, 2004; Knight et al., 2009; Wood, Holland, Hughes, & Goulson, 2015). To match the nature of our sampling (relatively intensive sampling in continuous space), we took an approach to deriving this estimate that differed from those of previous studies in which workers were sampled from spatially independent habitat patches (Chapman et al., 2003; Darvill et al., 2004; Knight et al., 2009; Wood, et al., 2015). In our approach, we used an “Abundance Coverage Estimator” (ACE) originally devised to quantify species richness (Chiu, Wang, Walther, & Chao, 2014) to estimate the number of unsampled colonies using resampling (Supporting information Text S2). This approach is justified because estimating total species richness and colony numbers are statistically directly analogous. Moreover, the ACE was specifically devised for estimating richness in communities of species that vary in abundance and hence detectability, and gives a conservative estimate of total species number (Chiu et al., 2014). With this approach, estimates of the total number of colonies, incorporating unsampled colonies, were produced using the R package “vegan” (Oksanen et al., 2014). For calculating nesting density, the estimated total number of colonies was then divided by the area of sampling, plus the area of the buffer around its periphery defined by the mean worker foraging distance.

To estimate the lineage survival rate between the two study years (Carvell et al., 2017), we used COLONY v2 to reconstruct the genotypes of the mothers of the workers sampled in 2015. We filtered these genotypes to include only loci where the genotype was known with a probability ≥0.8, yielding a set of “inferred queen genotypes.” A further colony assignment in COLONY v2 using identical settings, including priors for queen mating frequency, but with the inferred 2015 queen genotypes and the genotypes of the 2014 workers, was then used to test whether the queens that founded the colonies sampled in 2015 were full or half‐sisters of the workers sampled in 2014. The assignment of one of these queens as a sister of a colony of 2014 workers with a probability ≥0.8 was taken to indicate that both belonged to a lineage surviving across years, that is, that a daughter queen produced by the 2014 colony had founded the 2015 colony. Lineage survival rate was then estimated as the fraction of 2014 colonies that contributed to a colony lineage surviving until 2015.

### 2.9 Isolation by distance

Following Dreier et al. (2014), we used the inferred queen genotypes described above to investigate the fine‐scale spatial genetic structuring of *B. hypnorum* nests within the study area. First, based on the inferred queen genotypes, we estimated pairwise relatedness between all inferred colony queens with COANCESTRY (Wang, 2011). Second, using the reconstructed positions of the nests of the inferred queens, we used a linear model to test whether relatedness of these queens covaried with the geographic distance between their nests (isolation by distance).

## 3 RESULTS

### 3.1 Hardy–Weinberg equilibrium, null allele frequencies and linkage disequilibrium

After correction for multiple comparisons, three of the 20 polymorphic loci significantly deviated from HWE across both 2014 and 2015 worker samples. A further four loci significantly deviated from HWE across 2015 worker samples only (Supporting information Table S4). In addition, six of the 20 loci returned estimated null allele frequencies greater than 0.1. Of these, three were the same loci that deviated from HWE in both years and so these six loci were not used for colony assignment (Supporting information Table S4). No pairwise combination of loci showed significant evidence for linkage disequilibrium after correction for multiple comparisons (400 pairwise comparisons, corrected alpha value = 0.000125, minimum *p* value = 0.007). These results led to 14 of the 20 loci being retained for further analyses (Supporting information Table S4) and, for these loci, 645 workers were successfully genotyped at a median of 11 (interquartile range, 10–14) loci per worker.

### 3.2 Mating frequency of queens

None of the 44 collected queens were assigned as full sisters with a probability of greater than 0.8 (range, 0.001–0.731), and only two collected queens were assigned as likely half‐sisters (probability, 0.832). Therefore, the estimates of mating frequency were conducted using queen genotypes that were largely independent of one another.

For the estimation of queen mating frequency alone, all 20 polymorphic loci were used. This was because, in this analysis, all inference depended on simulated haploid males and so would not have been affected by deviation from HWE. In addition, the presence of null alleles is likely to have had only a small effect on our estimates of the mating frequency of each queen, as these estimates were based on multiple loci, the majority of which had very low estimated frequencies of null alleles (Supporting information Table S4). On this basis, multi‐locus genotypes were obtained for all of the 44 sperm samples, at a median (range) of 17 (6–19) loci (Supporting information Figure S3). None of the dissection or reaction negative controls contained any allelic peaks.

The results of Simulation 1 showed that, across the sampled queens, the mean expected number of loci at which a queen genotype would be matched by a combination of two random male mates by chance was 6.03. This gave a locus‐level expected rate of matching of 0.369 (standard deviation, 0.075), which in turn gave a critical value of 0.520. This meant that, if a queen's alleles were found in the genotype of the sperm taken from her spermatheca at more than 52% of the loci, then it is unlikely that they were genuinely shared and it is more likely they arose from contamination (Supporting information Figure S4a). For all but one of the sperm samples, the observed rate of matching was above the critical value (Supporting information Figure S4b). Therefore, it was assumed that all of the sperm samples may have been contaminated with their corresponding queen's DNA. Hence, where both of the queen's alleles were present at a locus in the sperm sample, they were inferred to be contaminants.

**Figure 1:**
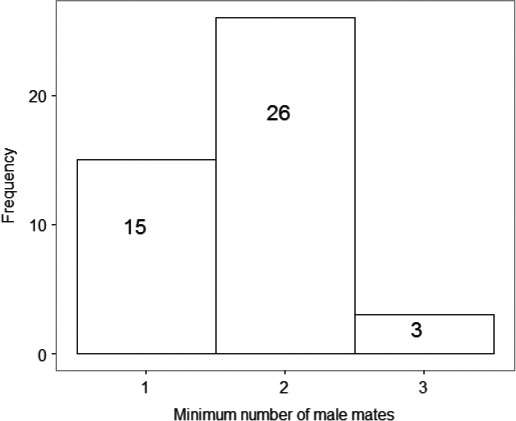
The frequency distribution of the minimum number of male mates (mating frequency) of 44 *Bombus hypnorum* queens, estimated from the maximum number of non‐queen microsatellite alleles, supported by more than one locus, present in sperm samples dissected from the queens' spermathecae

Counting only those alleles in the sperm genotypes that were not inferred to be contaminants for each queen indicated that of 44 queens, 34% (15/44) queens were singly mated and 66% (29/44) were mated twice or three times, with a mean (range) minimum mating frequency of 1.7 (1–3) (Figure 1). This is a conservative estimate of the actual mean mating frequency as the power to count further males is dependent on both the queen's and the males’ genotypes (see Discussion). The results of Simulation 2 indicated that, as an estimate of the frequency at which queens mate multiply (i.e. once vs. twice or more), our methodology is likely to be accurate. Only 1.2% of doubly mated queens were likely to be have been miscounted as singly mated. Triply‐ and quadruply‐mated queens were even less likely to have been miscounted as singly mated, with the estimated proportion of queens in which this would have occurred being 0.02% and 0.0002%, respectively (Supporting information Table S5). The method becomes less accurate and more likely to underestimate mating frequency as the true number of male mates rises. For example, the simulation shows that 22% of triply‐mated queens would be counted as only doubly mated. This meant that it was not possible to determine the true underlying frequency distribution of levels of queen multiple mating. However, it was possible to estimate the maximum number of mates that a queen may have had in the sample of 44 queens as the largest number of simulated “true” males that were likely to have been miscounted as the maximum observed number (i.e. 3). This indicates a maximum likely mating frequency of 5, since 6 true males would have been counted as 4, 5 or 6 observed males in 95% of cases (Supporting information Table S5).

### 3.3 Colony assignment

Initial runs of the COLONY v2 analysis without using a priori information on the queen mating frequency produced an estimate of the average number of worker representatives of a patriline in the sample of 1.44. This estimate of *n* and the estimate of queen mating frequency (*m* = 1.7) were used as prior values of the estimates of sampled sizes of maternal and paternal sibships (i.e., *mn* = 1.7 × 1.44 and *n* = 1.44, respectively) in the COLONY v2 analysis. In this analysis, 528 of the 645 workers were assigned to 78 colonies with a probability greater than 0.8. Sixteen of these assigned colonies were rejected as they had more than five patrilines (range, 6–8), leaving 62 colonies (hereafter, “accepted colonies”), 34 of which were sampled in 2014 and 28 in 2015. The pairwise distances between the sampling locations of full sisters were not significantly different from those of half‐sisters (*t*‐test not assuming equal variances, *t* = −1.53, *df* = 11.97, *p* = 0.152), which suggests that unrelated workers had not been erroneously over‐assigned as half‐sisters to the reconstructed colonies. In total, 189 and 89 distinct colonies were sampled in 2014 and 2015, respectively, comprising the 62 accepted colonies, plus colonies represented by just one sampled worker.

### 3.4 Colony‐specific worker foraging distance

**Figure 2:**
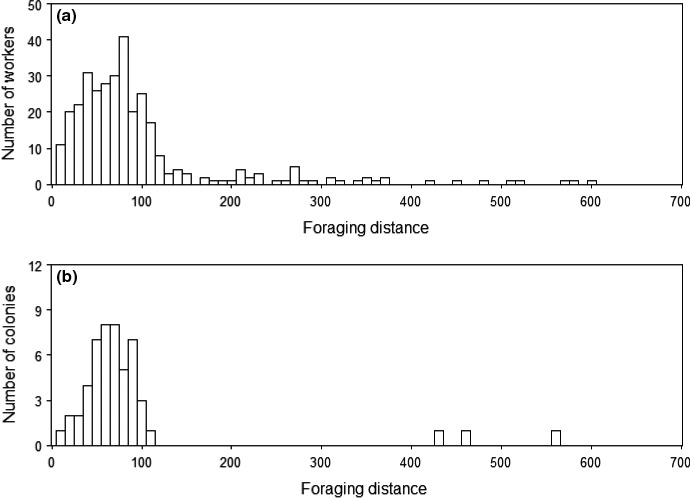
Foraging distances (m) of workers of *Bombus hypnorum *sampled in 2014 and 2015. Frequency distribution of (a) individual foraging distances of workers (*n* = 347 workers) and (b) estimated colony‐specific foraging distances averaged over all sampled workers in accepted colonies (*n* = 62 colonies containing 347 workers, each containing at least two workers). 34 colonies were sampled in 2014 and 28 in 2015

The mean (range) colony‐specific worker foraging distance over the 62 accepted colonies was 103.6 m (13.5–460.6 m) (Figure 2). The maximum individual worker foraging distance was 601 m. Colony‐specific worker foraging distances were not significantly different between years (2‐sample *t*‐test: *t* = −0.969, *df* = 56.1, *p* = 0.338). The pairwise distances between unrelated workers covered the range of possible distances permitted by the sampling area, while those of full and half‐sisters did not, suggesting the estimates of colony‐specific worker foraging distance were not constrained by the sampling area extent (Supporting information Figure S5).

### 3.5 Nesting density and lineage survival

From the ACE analysis, the total numbers of colonies present in the sampling area were estimated to be significantly higher in 2014 (1,244 colonies: 95% CI, 1,204–1,283) than in 2015 (350 colonies: 95% CI 329–372; Table 1). These values yielded estimated nesting densities of 2.56 and 0.72 colonies ha^−1^ in 2014 and 2015, respectively (Table 1).

**Table 1:** Estimates of the number and density of *Bombus hypnorum *colonies present in and around the 2 × 2 km sampling area by year of sampling. (a) Estimates of total colony numbers and overall densities. Colonies detected, number of different colonies workers were assigned to; Estimated number of colonies, detected colonies plus estimated number of undetected colonies using an “abundance coverage estimator” (Chiu et al., 2014), standard error in parentheses; Nesting density, colonies per hectare, standard error in parentheses, i.e. number of colonies divided by area of sampling area (400 ha) plus area of buffer within the mean colony‐specific worker foraging distance (103.6 m) of the periphery of the sampling area (86.25 ha); Sample completeness, proportion of estimated number of colonies that were sampled. (b) Number of colonies detected represented by differing numbers of workers. Singletons, number of colonies represented by one worker; Doubletons, number of colonies represented by two workers; Three or more, number of colonies represented by three or more workers; Maximum, largest number of workers representing a single colony

Year	Colonies detected	Estimated number of colonies	Nesting density (*SE*), in colonies ha^−1^	Sample completeness
(a)				
2014	189	1244.21 (20.07)	2.56 (0.05)	0.15
2015	91	350.38 (10.86)	0.72 (0.03)	0.25

Year	Singletons	Doubletons	Three or more	Maximum
(b)				
2014	158	2	29	14
2015	65	3	23	18

Fifteen of the 189 colonies sampled in 2014 had one or more of the 2015 mother queens assigned to them based on the inferred queen genotypes, which suggests a lineage survival rate of 0.07 (i.e. 15/189) between 2014 and 2015.

### 3.6 Isolation by distance

**Figure 3:**
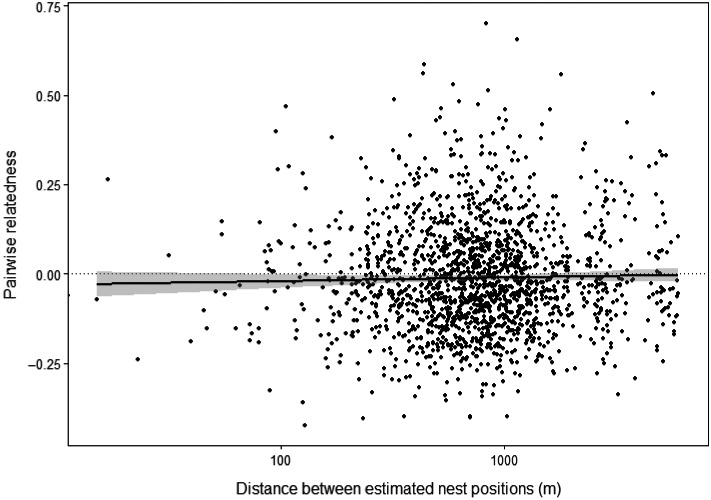
Relationship between pairwise relatedness of 62 *Bombus hypnorum* colony queens, whose genotypes were constructed from worker sibships, and distance between the estimated positions of their nests, on a log._10_ transformed scale, in a suburban 2 × 2 km study area in Norwich, UK. Black line, regression equation (*y* = [−1.63 × 10^−2^] + [3.94 × 10^−6^]*x*); gray ribbon, 95% confidence interval; dotted line, null hypothesis (*y = *0). The slope is not significant (*F*_1,1709_ = 1.173, *p* = 0.279, *R* ^2^ = 0.0007)

The relationship between pairwise relatedness of the inferred colony queens and the geographical distance between the estimated positions of their nests was not significant (*F*_1,1709 = _1.173, *p* = 0.279, *R* ^2^ = 0.0007; Figure 3). Therefore, at the fine spatial scale studied, there was no evidence for genetic structuring resulting from isolation by distance.

## 4 DISCUSSION

In this study, we used molecular methods to investigate the spatial ecology of an insect pollinator (*Bombus hypnorum*) that has recently undergone a rapid range expansion. Our results show that the mean colony‐specific worker foraging distance of *B. hypnorum* in a landscape typical of those in the southern UK was 103.6 m. *B. hypnorum *appears to nest at high densities that vary greatly from year to year, with estimated densities of 2.56 and 0.72 colonies per hectare in 2014 and 2015, respectively. The between‐year lineage survival rate was estimated to be 0.07 and there was no evidence of fine‐scale isolation by distance. Our results also suggest that 34% of *B. hypnorum *queens mated with just one male, that queens overall mated with a mean of 1.7 males and that individual queens may mate with up to five males.

Almost all previous studies quantifying worker foraging distances within bumble bee populations have found them to be greater than our estimated value for *B. hypnorum* (103.6 m). For example, Redhead et al. (2016) used a worker sampling protocol very similar to the one in the current study to quantify the colony‐specific foraging distances of five UK bumble bee species (*B. hortorum, B. lapidarius, B. pascuorum, B. ruderatus *and *B. terrestris*) in a lowland agricultural landscape, and found the range of species means to be 272–551 m. A study of four North American alpine bumble bee species reported very short worker foraging distances of 25–110 m (Geib, Strange, & Galen, 2015). However, Geib et al. (2015) used four discrete sampling sites each 0.79 ha in area, with minimum separation of 255 m, a sampling design that may have led the reported worker foraging distances to be underestimates. In addition, it needs recognizing that nearly all estimated worker foraging distances in bumble bees, including the present one for *B. hypnorum*, come from studies of single populations, and combining different estimates for single species from different studies shows that worker foraging distance may vary considerably between populations and hence be locality‐specific (Charman, Sears, Green, & Bourke, 2010).

While the mean *B. hypnorum* worker foraging distance was found to be notably low, the maximum individual worker foraging distance of 601 m was similar to previous estimates in other species. For example, Darvill et al. (2004) estimated a maximum foraging distance in *B. terrestris* of 625 m and Knight et al. (2005) estimated maximum foraging distances in *B. pascuorum*, *B. pratorum*, *B. lapidarius* and *B. terrestris *of 449, 674, 450 and 758 m, respectively. These values, combined with the strong evidence that bumble bee foraging distances are plastic (Carvell et al., 2012; Jha & Kremen, 2013; Pope & Jha, 2018; Redhead et al., 2016), suggest that, in the study population of *B. hypnorum*, a high density of foraging resources is driving the short‐range foraging observed on average. However, the observation that some *B. hypnorum *workers in our study foraged over larger distances similar to those reported for other species indicates that the low mean foraging distance estimated in the present study is not an autecological characteristic. Rather, it indicates that, while capable of foraging at the longer distances reported for other species, *B. hypnorum* workers in the study population are able to forage more profitably by travelling shorter distances to forage patches. Hence, in the range‐expanding *B. hypnorum*, our results support the prediction of short worker foraging distances that stems from the emerging synthesis (see Introduction) by which bumble bee population dynamics are linked to the local density of foraging resources (Carvell et al., 2017; Dicks et al., 2015; Redhead et al., 2016). Note that this inference does not assume that the range of *B. hypnorum *is still expanding throughout the UK or that *B. hypnorum'*s range expansion has been more rapid than others in *Bombus *species. Rather, it relies on the assumption that the demographic expansion required to underpin a range expansion implies ecological conditions rich in foraging resources for the focal species.

While our estimates of worker foraging distance could be subject to some biases, it is unlikely that these biases account entirely for the difference between our estimates and the higher estimates for other species’ foraging distances. In addition, it is worth noting that our estimates of worker foraging distance did not differ significantly across years, despite the wide difference in numbers of sampled workers and estimated nesting density across years (Table 1). Nonetheless, a possible source of bias is over‐assignment of workers, as polyandry in the study *B. hypnorum* population could have led to workers being erroneously assigned to colonies more frequently than in other studies in which queens are monandrous. In particular, relatedness among half‐siblings (0.5) is lower than that of full siblings (0.75), making the colony assignments of half‐sisters less certain. However, this factor would have biased the estimation of worker foraging distance upwards, since a worker assigned to a colony in error is more likely to have been sampled further away from the estimated nest position than a worker that had actually originated from the colony. Regardless, since half‐sisters were not sampled at significantly greater pairwise separation distances than full sisters, it is unlikely that overassignment had any effect on the estimates of worker foraging distance. Equally, underassignment of workers to colonies cannot be excluded, but again it is unlikely that this biased the estimates of worker foraging distance. This is because erroneous non‐assignment of a worker to its colony is likely to have occurred at random with respect to the worker's position in the distribution of worker foraging distances.

Our estimates of nesting density are notable as the estimate for 2014 is very high compared to estimates for other *Bombus *species (Chapman et al., 2003; Charman et al., 2010; Darvill et al., 2004; Knight et al., 2005) and there is large variation between the two sampled years. High nesting density could stem from the artificial cavities favoured by *B. hypnorum* for nesting being common within the suburban study landscape (Crowther et al., 2014). Large between‐year variation in nesting density points to the possibility of large demographic fluctuations in *B. hypnorum* numbers at a local scale. Such fluctuations stemming partly from variation in annual weather conditions have been described in the annual eusocial wasp *Vespula vulgaris*, which, moreover, showed similar population dynamics in its native and introduced ranges (Lester, Haywood, Archer, & Shortall, 2017). Confirming such a phenomenon in *B. hypnorum*, or a significant role for stochasticity in its temporal population dynamics, would be a significant finding as most previous studies of *Bombus *nesting density involved sampling in only one year. However, such confirmation would require further study across multiple populations and years. Since the *Bombus* species in previous studies of nest density are monandrous and therefore less likely to be subject to underassignment of workers to colonies, it is also possible that the apparently far greater nest density of *B. hypnorum* is at least partly an artifact. This is because underassignment is more likely to produce singletons, i.e. workers from colonies with only one sampled member, which represented 83.6% and 71.4% of colonies detected in 2014 and 2015, respectively (Table 1). This is likely to inflate the estimate of the number of unsampled colonies and hence of total colony number (Chapman et al., 2003; Chiu et al., 2014; Darvill et al., 2004; Knight et al., 2009; Wood et al., 2015). A related possibility is that previous studies that assumed monandry in the study species of *Bombus* have done so incorrectly, if in fact these species exhibit low frequencies of polyandry. Nonetheless, our evidence that *B. hypnorum* can attain very high nesting densities is consistent with its range expansion being associated with high population‐level productivity.

The estimated lineage survival rate between years in *B. hypnorum* (0.07) was low compared to the only other estimate of site‐level *Bombus *lineage survival, which was 0.24 (i.e. 0.41 × 0.59, using modelled apparent survival rates) across three established UK *Bombus* species from a site in southern England (Carvell et al., 2017). However, differences between the studies make it difficult to compare these rates. Since both studies exclude lineages of queens that left the study areas, the estimates of lineage survival would be most comparable across sites of similar sizes, yet the sampling area of the current study (400 ha) was smaller than that of Carvell et al. (2017), at 1,950 ha. Furthermore, Carvell et al. (2017), by using data from more colony cycle stages, were able to adjust their estimate for imperfect rates of lineage recapture.

The finding that the *B. hypnorum* population exhibited no significant genetic isolation by distance at the scale studied matches the findings of similar analyses of other *Bombus *species (Dreier et al., 2014). It suggests that, as in these other species, queens of *B. hypnorum* do not tend to found nests near their natal nest. A lack of genetic structure at this scale (2 × 2 km) is consistent with gene flow and genetic mixing at larger scales, although determining whether isolation by distance is absent at larger scales in *B. hypnorum* would require data from multiple populations.

We found that *B. hypnorum* queens in the study population had higher levels of polyandry than *B. hypnorum *queens collected from continental Europe. Across studies from continental Europe with sample sizes of 10 or more queens, the mean queen mating frequency ranged from 1 to 1.5 (Brown, Schmid‐Hempel, & Schmid‐Hempel, 2003; Paxton et al., 2001; Schmid‐Hempel & Schmid‐Hempel, 2000). Combined, these studies and the present findings support the conclusion that the mating frequency of *B. hypnorum *queens varies geographically (Brown et al., 2003). Polyandry might facilitate range expansion by increasing the effective population size at newly‐colonized sites. This is because a given number of colonizing queens that are multiply mated will, on average, have more genetic variation represented in the stored sperm of their male mates than the same number of singly mated queens. Most *Bombus *species exhibit single queen mating (Schmid‐Hempel & Schmid‐Hempel, 2000). However, several North American species of the subgenus *Pyrobombus*, to which *B. hypnorum* belongs, have also been found to mate multiply. Specifically, queens of *B. bimaculatus*, *B. impatiens*, *B. mixtus* and *B. ternarius *were found to mate with up to 2, 3, 4 and 2 males, respectively (Payne, Laverty, & Lachance, 2003). Hence, multiple mating at variable rates appears to be a feature of the subgenus *Pyrobombus* and, as yet, there is no evidence that the higher mating frequency of *B.* *hypnorum* in the UK either contributes to, or is a consequence of, the UK range expansion.

In conclusion, we have applied molecular methods to elucidate some basic ecological parameters for *B. hypnorum* population within its recently‐colonized UK range. This is the first time that all of these parameters have been estimated for a rapidly range‐expanding *Bombus *species (Woodard et al., 2015). In addition, our findings support the hypothesis that range expansion, population‐level productivity and short worker foraging distances are associated with one another and, moreover, characteristic of the expanding UK *B. hypnorum* population.

## AUTHOR CONTRIBUTIONS

Andrew Bourke, Claire Carvell, Liam Crowther, David Richardson and David Wright designed the study. LC conducted the field and laboratory work. LC analyzed the data. LC wrote the manuscript. AB, CC, DR, and DW contributed comments and edits to manuscript drafts.

## Data Availability

All of the data associated with this manuscript is archived on Figshare. (https://doi.org/10.6084/m9.figshare.7284803).
