# Supplementary material for: Spatial ecology of a range‐expanding bumble bee pollinator
Source: Ecol Evol. 2019 Jan 8;9(3):986–97. doi: 10.1002/ece3.4722 (PMC6374649; doi:10.1002/ece3.4722)
Supplement: Supplementary file 1 [file ECE3-9-986-s001.doc]

**Appendices**

**Supplemental Methods**

**Text S1 *Spermathecal dissection***

While still frozen, the gaster (major part of the abdomen) was cut from the rest of the body and each abdominal sternite was cut using micro-dissection scissors such that an incision ran centrally along the full length of the gaster, on the ventral side. Care was taken not to incise the still-frozen soft tissues beneath. The cut sternites were then removed by manipulating them with forceps to tear them free from their corresponding tergites (Appendices: Figure S2a). By this stage the soft tissues exposed by the removal of the sternites had usually thawed, and the gut was teased out using forceps without displacing the ovaries. The spermatheca was then visually located. If the ovaries had not been displaced it could be found attached to the junction of the ovaries at the bursa, to which the spermatheca is attached by a short duct. If the sting was in a retracted position, the sting was extended by pressing it with a needle to expose the bursa and spermatheca. The spermathaca was held with fine forceps by the sperm duct and torn from its attachment to the bursa (Appendices: Figure S2b).

**Text S2 *Nesting density estimation***

Previous studies of bumblebee nest density using assignment to colonies based on microsatellite markers have taken two different approaches to estimating the number of un-sampled nests. One method is to use a truncated Poisson distribution to estimate how many colonies were represented in the sample by zero workers, which assumes all colonies are equally detectable (Chapman, Wang, & Bourke, 2003; Darvill, Knight, & Goulson, 2004; Knight et al., 2009). The second method uses a mark-recapture approach in which colonies can belong to two groups with two different detection probabilities (the two-innate-rates-model; Wood et al. 2015). The present study differed in its sampling strategy from these studies, which sampled workers from discrete sites across a landscape. By contrast, in the present study we sampled intensively and continuously across the landscape (via a grid design), with the aim of detecting as many of the colonies present as possible. In addition, it is likely that, instead of there being one or two constant rates of colony detection, actually the detectability of every colony is different. This is because many traits that are presumably associated with the detectability of a colony, such as colony size, vary across colonies. It follows that, with greater sampling intensity, the sampled colonies will present a greater range of detectability and hence that the assumptions of the previous studies will be less applicable.

We therefore applied a method originally devised for estimating species richness from samples that vary in completeness, specifically an ‘abundance coverage estimator‘, hereafter ACE (Chiu, Wang, Walther, & Chao, 2014) This represents the first application of this approach to estimating bumblebee nesting density, and is justified because it is statistically directly analogous to the established use of the ACE for estimating species richness. Moreover, the ACE was specifically devised for estimating species richness in communities of species that vary in abundance and hence in detectability and gives a conservative estimate of the total number of species (Chiu et al., 2014). The ACE produces an estimate of species richness based on counts of the individuals detected, using resampling to estimate the ‘completeness’ of the sample, i.e. the proportion of all the species that have been detected. Hence, it is robust to differences in sample size. In the current study we treated each colony as a ‘species’ and the number of workers that were sampled from that colony as its individual counts. On this basis, estimates of the number of colonies were produced using the R package ‘vegan’ (Oksanen et al., 2016). Sampled workers that were not assigned to colonies were assumed to belong to distinct colonies that were only represented by one sampled worker. For calculating nesting density, the estimated number of colonies was then divided by the area of sampling plus the area of the buffer around its periphery defined by the mean worker foraging distance.

**Table S1. Sites used for collection of *Bombus hypnorum* queens in Norfolk, UK**

| Site | Latitude | Longitude |
| --- | --- | --- |
| Whitlingham Country Park | 52° 37' 0.4008'' | 1° 14' 20.2776'' |
| Drayton | 52° 41' 12.1992'' | 1° 12' 37.8468'' |
| University of East Anglia | 52° 37' 5.358'' | 1° 14' 3.0516'' |
| Spixworth | 52° 41' 30.1812'' | 1° 19' 1.614'' |
| Hethersett | 52° 36' 1.998'' | 1° 10' 40.2384' |

**Table S2. Microsatellite loci tested on *B. hypnorum* but excluded from the current study. Marker, locus identifier designated by authors that originally identified the locus (Estoup et al., 1995; Reber-Funk et al., 2005; Stolle et al., 2009); Reason for exclusion, whether PCRs produced either no amplicons or the amplicons were monomorphic size fragments; Size, amplicon length in base pairs; n, number of *B. hypnorum*** workers tested; n/a, not applicable

| Marker | Reason for exclusion | Size | n |
| --- | --- | --- | --- |
| BL13 | Monomorphic | 163 | 20 |
| BL15 | Failed to amplify | n/a | 15 |
| BTMS0126 | Failed to amplify | n/a | 15 |
| BTMS0151 | Failed to amplify | n/a | 15 |

**Table S3. Conditions for multiplex PCRs to co-amplify microsatellite fragments from *Bombus hypnorum* template DNA for 20 loci in three multiplexes (A, B, C). Marker, microsatellite locus identifier; dye, fluorescent molecule added to 5’ end of forward primer; size ran**ge, min – max size of amplicons in base pairs; primer concentration, molar concentration of each oligonucleotide for forward and reverse priming in final reaction volume (2 μl).

| Multiplex | Marker | Dye | Size range | Primer concentration |
| --- | --- | --- | --- | --- |
| A | B131 | 6-FAM | 118 - 130 | 0.08 |
|  | B132 | HEX | 159 - 179 | 0.50 |
|  | BL03 | 6-FAM | 144 - 160 | 0.20 |
|  | BT26 | HEX | 98 - 110 | 0.08 |
|  | BTMS0125 | ATTO-550 | 110 - 149 | 0.20 |
|  | BTMS0132 | HEX | 134 - 146 | 0.20 |
| B | B10 | 6-FAM | 178 - 200 | 0.40 |
|  | B11 | 6-FAM | 158 - 164 | 0.20 |
|  | B121 | ATTO-550 | 153 - 208 | 0.35 |
|  | B96 | 6-FAM | 243 - 255 | 0.50 |
|  | BT05 | HEX | 153 - 162 | 0.12 |
|  | BTMS0033 | HEX | 201 - 204 | 0.30 |
|  | BTMS0056 | HEX | 254 - 256 | 0.20 |
|  | BTMS0057 | HEX | 104 - 113 | 0.08 |
| C | BL01 | ATTO-550 | 134 - 148 | 0.20 |
|  | BL08 | HEX | 145 - 149 | 0.35 |
|  | BT10 | 6-FAM | 118 - 124 | 0.10 |
|  | BTERN01 | HEX | 114 - 127 | 0.08 |
|  | BTERN02 | 6-FAM | 157 - 179 | 0.25 |
|  | BTMS0083 | 6-FAM | 277 - 306 | 0.10 |

**Table S4. Summary of population-genetic data by locus for sampled *Bombus hypnorum*** individuals. k, number of alleles; N, number of individuals for which a genotype was successfully obtained at that locus; H Obs, observed frequency of heterozygotes; H Exp, expected frequency of heterozygotes; HWE 2014, HWE 2015, result of Bonferroni-corrected test of the null hypothesis that the locus is not significantly out of Hardy-Weinberg equilibrium for workers sampled in 2014 or 2015, respectively; p 2014, p 2015, p value for corresponding HWE hypothesis test calculated using chi-squared test for associations; F(Null), estimated frequency of null alleles across all workers; Action, decision on use of locus in further population genetic analyses.

| Locus | Queens |  |  |  |  | Workers |  |  |  |  |  |  |  |  |  |
| --- | --- | --- | --- | --- | --- | --- | --- | --- | --- | --- | --- | --- | --- | --- | --- |
|  | k | N | H Obs | H Exp |  | k | N | H Obs | H Exp | HWE 2014 | p 2014 | HWE 2015 | p 2015 | F(Null) | Action |
| B10 | 7 | 44 | 0.659 | 0.67 |  | 9 | 595 | 0.615 | 0.615 | TRUE | 0.132 | TRUE | 0.855 | 0.0013 | Retain Marker |
| B11 | 4 | 44 | 0.568 | 0.668 |  | 4 | 584 | 0.62 | 0.644 | TRUE | 0.196 | TRUE | 0.024 | 0.0203 | Retain Marker |
| B121 | 5 | 44 | 0.523 | 0.744 |  | 5 | 434 | 0.576 | 0.648 | TRUE | 0.321 | TRUE | 0.034 | 0.0569 | Retain Marker |
| B131 | 3 | 44 | 0.432 | 0.452 |  | 5 | 605 | 0.364 | 0.403 | TRUE | 0.04 | FALSE | < 0.001 | 0.0543 | Retain Marker |
| B132 | 6 | 44 | 0.477 | 0.501 |  | 7 | 309 | 0.469 | 0.635 | TRUE | 0.448 | FALSE | < 0.001 | 0.1586 | Drop Marker |
| B96 | 5 | 44 | 0.295 | 0.683 |  | 4 | 467 | 0.45 | 0.662 | FALSE | < 0.001 | FALSE | < 0.001 | 0.1928 | Drop Marker |
| BL01 | 5 | 44 | 0.25 | 0.592 |  | 8 | 328 | 0.46 | 0.722 | TRUE | 0.694 | FALSE | < 0.001 | 0.2343 | Drop Marker |
| BL03 | 5 | 44 | 0.477 | 0.526 |  | 9 | 582 | 0.498 | 0.491 | TRUE | 0.003 | TRUE | 0.543 | -0.0134 | Retain Marker |
| BL08 | 3 | 44 | 0.045 | 0.39 |  | 3 | 362 | 0.155 | 0.153 | TRUE | 0.645 | TRUE | 0.004 | -0.0086 | Retain Marker |
| BT05 | 5 | 44 | 0.636 | 0.599 |  | 4 | 605 | 0.57 | 0.581 | TRUE | 0.476 | TRUE | 0.226 | 0.0106 | Retain Marker |
| BT10 | 4 | 44 | 0.432 | 0.515 |  | 4 | 389 | 0.391 | 0.599 | FALSE | < 0.001 | FALSE | < 0.001 | 0.213 | Drop Marker |
| BT26 | 5 | 44 | 0.545 | 0.569 |  | 7 | 587 | 0.578 | 0.578 | TRUE | 0.047 | TRUE | 0.342 | 0.0037 | Retain Marker |
| BTERN01 | 4 | 44 | 0.25 | 0.4 |  | 7 | 392 | 0.156 | 0.567 | FALSE | < 0.001 | FALSE | < 0.001 | 0.5861 | Drop Marker |
| BTERN02 | 6 | 44 | 0.227 | 0.696 |  | 8 | 346 | 0.754 | 0.749 | TRUE | 0.615 | TRUE | 0.026 | -0.0085 | Retain Marker |
| BTMS0033 | 3 | 44 | 0.159 | 0.402 |  | 2 | 558 | 0.238 | 0.326 | TRUE | 0.004 | FALSE | < 0.001 | 0.1556 | Drop Marker |
| BTMS0056 | 4 | 44 | 0.136 | 0.172 |  | 3 | 568 | 0.164 | 0.158 | TRUE | 0.814 | TRUE | 0.824 | -0.0221 | Retain Marker |
| BTMS0057 | 5 | 44 | 0.614 | 0.592 |  | 5 | 613 | 0.618 | 0.65 | TRUE | 0.023 | TRUE | 0.722 | 0.0214 | Retain Marker |
| BTMS0083 | 8 | 44 | 0.682 | 0.755 |  | 6 | 281 | 0.598 | 0.694 | TRUE | 0.479 | FALSE | < 0.001 | 0.0754 | Retain Marker |
| BTMS0125 | 2 | 44 | 0.023 | 0.023 |  | 11 | 580 | 0.669 | 0.72 | TRUE | 0.034 | TRUE | 0.002 | 0.0337 | Retain Marker |
| BTMS0132 | 1 | 44 | 0 | 0 |  | 3 | 536 | 0.011 | 0.015 | TRUE | 0.005 | TRUE | 0.99 | 0.0986 | Retain Marker |

**Table S5. Results of Simulation 2 for estimating the queen mating frequency of *Bombus hypnorum*, i.e. probabilities of observing 1 – 9 males in sperm samples, given the simulated ‘true’ number of males. Forty-four *Bombus hypnorum* queens were genotyped at a median (range) of 17 (6 - 19) microsatellite loci. Population allele frequencies from 645 *B. hypnorum*** workers in the study population were used to simulate sperm genotypes based on 1 - 9 contributing males. Each queen genotype was paired with 10,000 simulated sperm genotypes for each of the 1 - 9 simulated values of the ‘true’ number of males. Observed number of males was then counted from the combined queen and sperm sample genotype, assuming that alleles shared with the queen were contamination.

|  | ‘True’ number of males | | |  |  |  |  |  |  |
| --- | --- | --- | --- | --- | --- | --- | --- | --- | --- |
| Observed number of males | 1 | 2 | 3 | 4 | 5 | 6 | 7 | 8 | 9 |
| 1 | 0.999852 | 0.011964 | 0.000225 | 2.27E-06 | 0 | 0 | 0 | 0 | 0 |
| 2 | 0 | 0.988036 | 0.223486 | 0.01652 | 0.001395 | 0.00013 | 6.82E-06 | 2.27E-06 | 0 |
| 3 | 0 | 0 | 0.776289 | 0.685734 | 0.22685 | 0.053093 | 0.012289 | 0.002914 | 0.000732 |
| 4 | 0 | 0 | 0 | 0.297743 | 0.718698 | 0.638714 | 0.363143 | 0.16508 | 0.069068 |
| 5 | 0 | 0 | 0 | 0 | 0.053057 | 0.303498 | 0.575214 | 0.655068 | 0.560993 |
| 6 | 0 | 0 | 0 | 0 | 0 | 0.004566 | 0.049136 | 0.173875 | 0.35263 |
| 7 | 0 | 0 | 0 | 0 | 0 | 0 | 0.000211 | 0.003055 | 0.016498 |
| 8 | 0 | 0 | 0 | 0 | 0 | 0 | 0 | 6.82E-06 | 7.95E-05 |
| 9 | 0 | 0 | 0 | 0 | 0 | 0 | 0 | 0 | 0 |


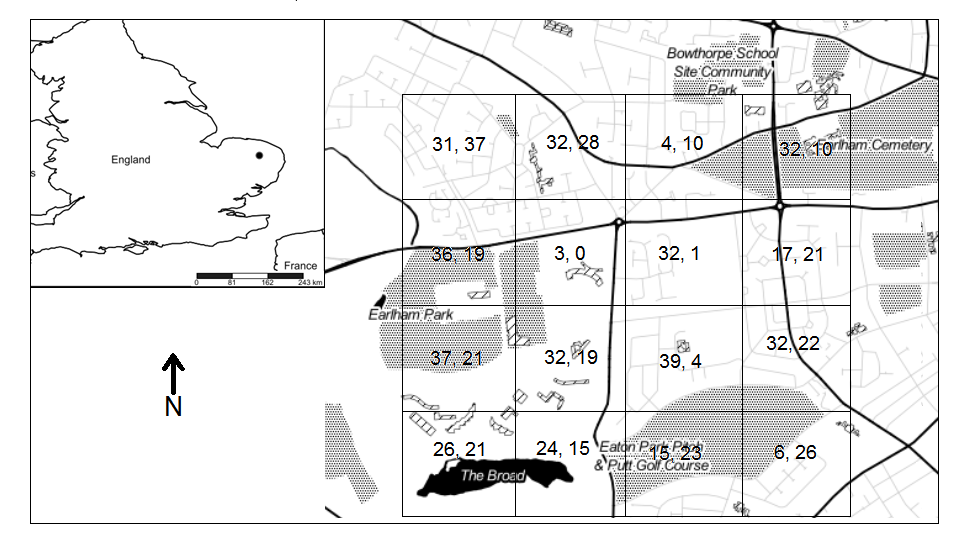


**Figure S1. The sampling area for *Bombus hypnorum* workers, a 2 x 2 km tetrad divided into sixteen 500 x 500 m sampling squares; numbers represent workers collected in 2014 and (after comma) 2015. Inset: Location of study site in eastern England, UK. The position of the sampling area’s southwestern corner was: 52°36′56.12″N, 001°14′00.39″E. Main map legend: Black, water; grey, parkland; white, suburbia and semi natural. Thick black lines, major roads; fine black lines, sampling square divisions; grey lines, minor roads. In total, 398 and 277 workers were collected in 2014 and 2015, respectively.**


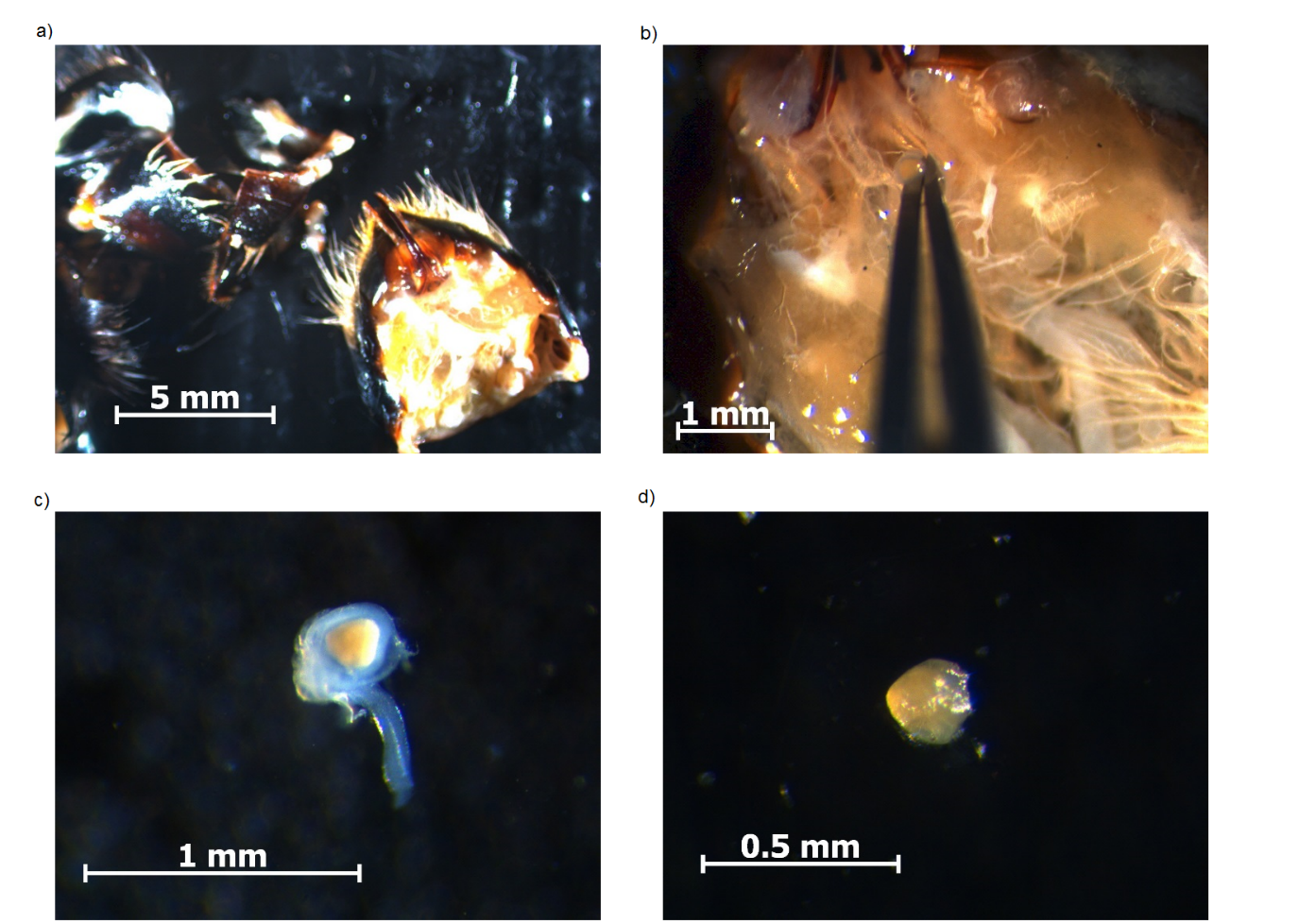


**Figure S2. Steps in the dissection of the spermatheca from a *Bombus hypnorum* queen. a) Removal of the queen's sternites; b) Isolation of spermatheca using fine forceps; c) spermatheca with sperm duct and glands suspended in droplet of distilled water; d) sperm mass isolated from spermathecal structures.**

**
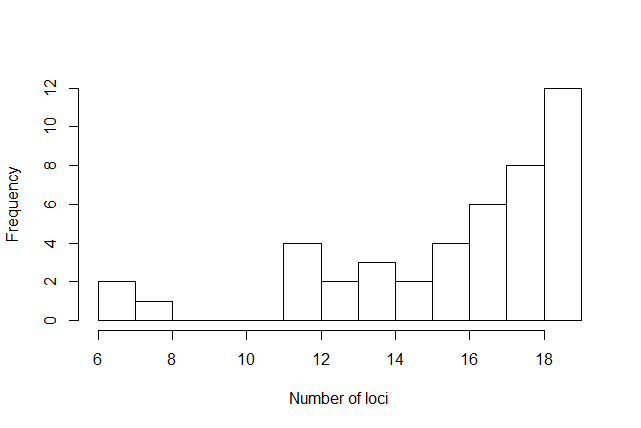
**

**Figure S3. Frequency distribution of the number of microsatellite loci available in paired queen and sperm samples to estimate the mating frequency of 44 *Bombus hypnorum* queens.**

**
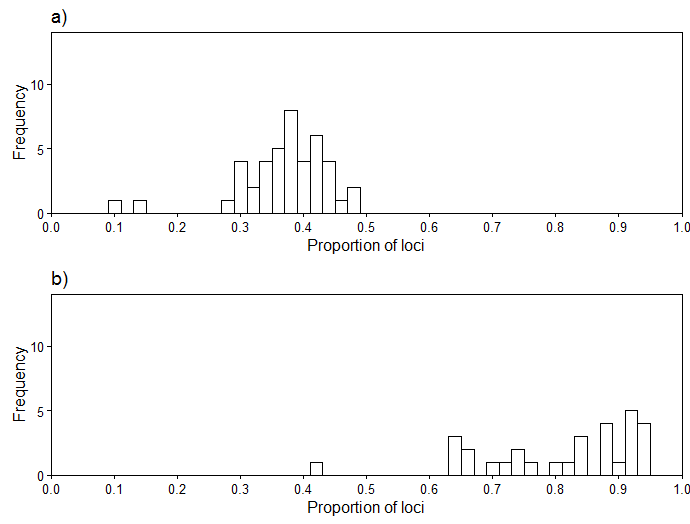
**

**Figure S4. Frequency distributions of the proportion of microsatellite loci at which a *Bombus hypnorum* queen’s alleles were found, for a given locus, in the genotype of the sperm sample dissected from her spermatheca (*n* = 44 queen and corresponding sperm samples). a) Expected distribution, assuming double mating, based on simulation of 10,000 random pairs of males drawn from population allele frequencies of 645 *B. hypnorum* workers; b) Observed distribution from actual sperm samples.**

**
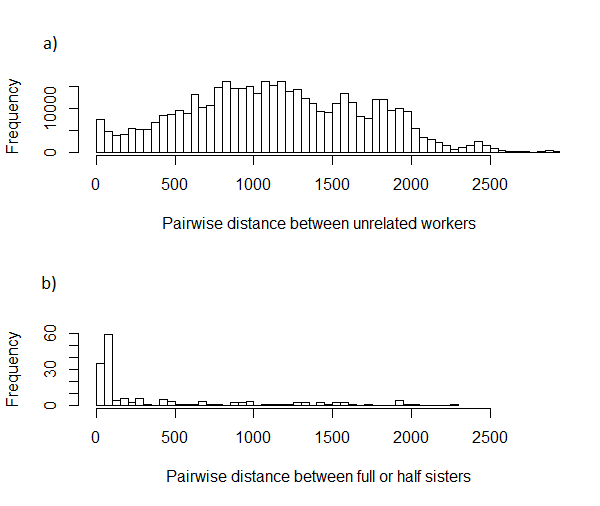
**

**Figure S5. Frequency distributions of the distance (m) between the sampling locations of pairs of: a) unrelated workers and b) full or half-sister workers.**
